# Draft Genome Sequences of Two Antimicrobial-Producing *Burkholderia* sp. Strains, MSh1 and MSh2, Isolated from Malaysian Tropical Peat Swamp Forest Soil

**DOI:** 10.1128/genomeA.01032-14

**Published:** 2014-10-09

**Authors:** Kuan Shion Ong, Yoong Kit Aw, Han Ming Gan, Catherine M. Yule, Sui Mae Lee

**Affiliations:** School of Science, Monash University Malaysia, Selangor, Malaysia

## Abstract

We report the draft genome sequences of two antimicrobial-producing isolates, *Burkholderia* sp. strains MSh1 and MSh2, which were isolated from tropical peat swamp forest soil. Putative genes related to different antimicrobial production have been annotated in both genome sequences.

## GENOME ANNOUNCEMENT

Extensive use of antimicrobials has led to an increase in infections caused by antimicrobial-resistant bacteria (ARB). The increase in mortality rates from infection coupled with limited treatment options has prompted the need for new antimicrobials (1). It is hypothesized that bacteria produce antimicrobial compounds in nutrient-poor and extreme environments to gain an advantage in competing for resources (2). Hence, a Malaysian tropical peat swamp forest was chosen as a potential location for new antimicrobial compounds due to its low nutrient level and low pH conditions (3). Two antimicrobial-producing bacteria were successfully isolated and identified as *Burkholderia* sp. using 16S rRNA gene analysis (4).

Both *Burkholderia* sp. strains MSh1 and MSh2 are oxidase positive, non–spore forming, Gram-negative bacteria isolated from tropical peat swamp forest soil from Southeast Pahang, Malaysia. Both were isolated due to their ability to produce antibacterial compounds that were active against several drug-resistant bacteria such as methicillin-resistant *Staphylococcus aureus* (MRSA) ATCC 700699 and vancomycin-resistant enterococci (VRE) ATCC 700802 with MIC values of the crude acetonitrile extract at 1.250 mg/mL and 0.313 mg/mL, respectively.

The genomic DNA of *Burkholderia* sp. MSh1 and MSh2 were isolated from a 2-day-old culture on nutrient agar using a GF-1 nucleic acid extraction kit (Vivantis, Malaysia) and subsequently converted into an Illumina-compatible next-generation sequencing library using Nextera XT (Illumina, San Diego, CA). The library was then sequenced on the Illumina MiSeq (150-bp paired-end reads) at the Monash University Malaysia Genomics Facility. The raw reads were trimmed and assembled *de novo* (default settings) using CLC Genomics Workbench 6 (CLC Bio, Denmark). The draft genome of *Burkholderia* sp. MSh1 was assembled into 172 contigs with 67.08% G+C content and a total length of 8,633,651 bp (*N*_50_ = 110,286 bp), while *Burkholderia* sp. MSh2 had 167 contigs with 67.13% G+C content and an accumulated length of 8,723,138 bp (*N*_50_ = 129,187 bp).

Using the NCBI Prokaryotic Genome Annotation Pipeline (PGAP), 6,963 coding DNA sequences (CDSs), 10 rRNAs, and 64 tRNAs were annotated for *Burkholderia* sp. MSh1, while 7,072 CDSs, 9 rRNAs, and 59 tRNAs were annotated for *Burkholderia* sp. MSh2. Several antimicrobial biosynthesis genes were predicted in the genome of both *Burkholderia* sp. MSh1 and MSh2, including polyketide cyclase (KFG96276 and KEZ06389), antibiotic biosynthesis monooxygenase (KFG97610 and KEZ06948), mitomycin antibiotic biosynthesis protein (KFG98191 and KEZ04328), colicin V production protein (KFG93602 and KEZ04863), and phenazine biosynthesis protein (PhzC/PhzF) (KFG92381 and KEZ02706).

Based on RAST, some of the known antimicrobials produced by *Burkholderia* species were absent, for instance, pyrrolnitrin (*B. pyrrocinia*), rhizobitoxin (*B. andropogonis*), and pyoluteorin (*B. cepacia*) (5–7). This indicates that the antimicrobial compounds produced by these two isolates might be new and hence have the potential to treat infection caused by antimicrobial-resistant bacteria. However, further purification and identification of the antimicrobial compounds are required.

### Nucleotide sequence accession numbers.

The draft genome sequences of *Burkholderia* sp. MSh1 and MSh2 were deposited in DDBJ/EMBL/GenBank under the accession numbers JPGL00000000 and JPGM00000000, respectively. The versions described in this paper are the first versions, JPGL00000000.1 and JPGM00000000.1.
